# Growth-promoting effects of arbuscular mycorrhizal fungus *Funneliformis mosseae* in rice, sesame, sorghum, Egyptian pea and Mexican hat plant

**DOI:** 10.3389/fmicb.2025.1549006

**Published:** 2025-04-28

**Authors:** Rong Zhou, Ali Raza, Jueping Song, Sara Janiad, Qian Li, Miaomiao Huang, Muhammad Ahmad Hassan

**Affiliations:** ^1^Sericulture Research Institute, Anhui Academy of Agricultural Sciences, Hefei, China; ^2^Anhui Province Key Laboratory of Crop Integrated Pest Management, School of Plant Protection, Anhui Agricultural University, Hefei, China; ^3^Department of Microbiology & Molecular Genetics, The Women University Multan, Multan, Pakistan; ^4^College of Resource and Environment, Anhui Agricultural University, Hefei, China

**Keywords:** arbuscular mycorrhizal fungi, plant growth, plant nutrition, growth promoter, physiological parameters

## Abstract

Excessive use and overreliance on chemical fertilizers threatens soil health and environmental sustainability, necessitating eco-friendly alternatives like arbuscular mycorrhizal fungi (AMF). The benefits of AMF are well-documented in staple crops, their effects on diverse species—particularly legumes and non-crop models under uniform conditions—remain underexplored, limiting their scalable adoption. This study evaluated *Funneliformis mosseae*’s role in enhancing growth, nutrient uptake, and stress resilience across five species: rice (*Oryza sativa*), sesame (*Sesamum indicum*), sorghum (*Sorghum bicolor*), Egyptian pea (*Sesbania sesban*), and the non-crop *Kalanchoe daigremontiana*. The pot-experiment was conducted in natural open-field conditions (e.g., ambient light, temperature, and humidity) and inoculated plants were analyzed for biomass yield, nutrient concentrations, and physiological parameters to evaluate *F. mosseae*’s efficacy as a sustainable growth promoter. Inoculation with *F. mosseae* significantly enhanced plant performance across all species. Rice exhibited a 43% increase in dry biomass, alongside 53% higher phosphorus uptake and 24.5% greater magnesium accumulation. Root development improved markedly, with sesame, sorghum, Egyptian pea, and Mexican hat plants showing root length increases of 66.7, 42.9, 35, and 33.3%, respectively. Biomass gains were consistent: Egyptian pea (29% fresh biomass, 33% dry), sesame (30% fresh, 39% dry), sorghum (36.6% total), and Mexican hat plant (31% fresh, 34% dry). Nutrient uptake surged systemically, including potassium (sesame: 42%, Egyptian pea: 17.8%), calcium (sesame: 54.5%, sorghum: 29.4%), and magnesium (Mexican hat plant: 32.4%, Egyptian pea: 22.5%). Physiologically, photosynthetic rates rose by 21.4–45% (highest in Egyptian pea), stomatal conductance improved by 23.3–71.4% (peak in sesame), and chlorophyll *a* and *b* levels increased by 30–39.1% and 44.4–150.8%, respectively, across species. These results suggested that *F. mosseae* could provide a sustainable, environment friendly substitute for chemical fertilizers, preparing for the future of agriculture, where ecological services such as crop productivity and soil fertility depend on mycorrhizas alongside conventional cultivation practices. Integrating AMF into agricultural systems offers a potential strategy for eco-friendly farming practices that are viable and secure for long-term food security and eco-sustainability.

## 1 Introduction

During the Green Revolution, global crop productivity increased owing to the widespread use of chemical fertilizers, pesticides, and other agricultural inputs. This also allowed the large-scale cultivation of crops and their use to serve a fast-growing population. Despite this, many environmental and agricultural challenges have been caused by overreliance on chemical inputs (John and Babu, 2021). Excessive fertilizers and pesticides have degraded soil health, disturbed ecosystems, and polluted water and air (Baweja et al., 2020). For instance, pesticide usage has doubled worldwide since 1990, with an annual 3.5 million tons (Sharma et al., 2019). However, these practices are not just an assault on biodiversity; they also undermine the long-term fertility of soils, making farmers increasingly dependent on ever-increasing chemical applications to produce yields (Shattuck et al., 2023).

As the sustainability of traditional agricultural practices is becoming a concern for researchers and farmers alike, eco-friendly alternative options are being discovered to mitigate these issues (Gamage et al., 2023; Shattuck et al., 2023). Plant growth-promoting microorganisms (PGPM) are the most important and have emerged as powerful allies in sustainable agriculture. Bacteria and fungi are microorganisms that enhance nutrient availability in the soil, facilitate plant growth, and decrease environmental stress (Malgioglio et al., 2022; Rehman et al., 2024). Arbuscular mycorrhizal fungi (AMF) are vital soil microorganisms that form symbiotic associations with approximately 80% of terrestrial plant species (Abrar et al., 2024). These fungi enhance plant nutrient uptake, primarily phosphorus and nitrogen, by extending their hyphal networks into the soil (Khan et al., 2024). Through this symbiotic relationship, AMF reduce dependence on chemical fertilizers and contribute to sustainable agricultural practices (Samuel and Veeramani, 2021). In turn, the fungi use photosynthates from the host plants as a source of carbon, which, together with polysaccharide-derived carbon, is given as carbon from photosynthates to the fungi, constituting a mutualistic relationship (Bennett and Groten, 2022; Tang et al., 2023; Wang et al., 2020).

Among AMF species, *Rhizophagus irregularis* and *Glomus monosporum* are widely recognized for their role in promoting plant growth (Chandwani et al., 2023). However, *Funneliformis mosseae* has also emerged as a promising AMF species due to its ability to significantly enhance biomass production and nutrient acquisition in various crops. It colonizes plant roots, facilitating better nutrient uptake and optimizing plant physiological performance (Samuel and Veeramani, 2021). *F. mosseae* also contributes to soil fertility by producing glomalin, a glycoprotein associated with soil aggregation and carbon retention (Dahiya et al., 2022; Agnihotri et al., 2021). Despite these advantages, the potential of *F. mosseae* as a biofertilizer remains underexplored, particularly in diverse crop species.

This research study investigated the role of *Funneliformis mosseae*—a single AMF strain—in enhancing growth, nutrient uptake, and stress resilience across five ecologically diverse plant species: rice (*Oryza sativa*), sesame (*Sesamum indicum*), sorghum (*sorghum bicolor*), Egyptian pea (*Sesbania sesban*), and the non-crop model Mexican hat plant (*Kalanchoe daigremontiana*). Unlike prior studies focusing on individual crops or mixed AMF consortia, our work isolates the specific contributions of *F. mosseae* under uniform experimental conditions, addressing a critical gap in understanding species-specific AMF interactions. By integrating primary nutrients (N, P, K) with secondary macronutrients (Ca, Mg) and advanced physiological markers (chlorophyll fluorescence, photosynthetic efficiency), this study provides a holistic assessment of AMF-mediated benefits. Furthermore, the inclusion of Mexican hat, a stress-tolerant non-agricultural species, extends AMF research beyond traditional crops, offering insights into ecological restoration. These findings advance sustainable agriculture by demonstrating how targeted AMF inoculation can reduce reliance on chemical fertilizers while enhancing productivity and resilience across diverse plant systems.

## 2 Material and methods

The plant materials used in this study included five species: rice, sesame, sorghum Egyptian pea, and Mexican hat plant. The distinct characteristics and potential to serve as agents of growth and productivity were the basis for choosing these plant species: *F. mosseae*. This study used the fungus provided by the Institute of Plant Protection and Agro-Products Safety, Anhui Academy of Agricultural Sciences, Hefei, China. Pot experimentation was carried out in 2024 at the experimental station of the Anhui Academy of Agricultural Sciences, Hefei, China. For the pot experimentation, soil was collected from the upper 0–20 cm layer. This depth corresponds to the root-active zone in agricultural soils, where nutrient availability and microbial activity are highest. The collected soil was air-dried, sieved (2 mm mesh), and homogenized before being mixed with sterile sand (2:1 v/v) to prepare the growth medium for pot filling. The yellow-brown soil (pH = 7.8) was collected from Anhui Academy of Agricultural Sciences and had the following physicochemical properties: organic matter 18.2 g/kg, nitrogen 122.7 mg/kg, phosphorus 24.8 mg/kg and potassium 142 mg/kg. Soils were prepared using the mixture, and then 2 kg was placed in each pot (rectangle shape with dimensions L × W × H = 20 cm × 10 cm × 7 cm) to meet optimal conditions for plant growth and fungal inoculation. The meteorological conditions during the experimentation duration are given in Table 1.

**Table 1:** The weather conditions during experimentation.

Month/Year	Average temperature (°C)	Average humidity (%)	Average precipitation (mm)
May-2024	22.40	65.20	4.70
Jun-2024	26.88	68.93	4.42
Jul-2024	29.30	80.8	16.98
Aug-2024	31.53	75	1.35
Sep-2024	29.6	68.33	2

### 2.1 Plant growth

The experiment was conducted using six replicates per treatment group. Care was taken to ensure that the rice, sesame, sorghum, and Egyptian pea seeds were cleaned to remove any source of contamination. The seeds were then washed with tap water and sterile distilled water to ensure no residue was left on the seeds. The seeds were immersed in 70% ethanol for 1 min and then 10 min in 3% sodium hypochlorite solution to eliminate any potential contamination of microbial type that was found adequate to achieve surface sterilization of seeds (rice, sesame, sorghum, and Egyptian pea) (Davoudpour et al., 2020). The seeds were then sterilized and rinsed five times with sterile distilled water to ensure that all chemical residues were removed (Si et al., 2022).

The sterilized seeds were initially sown in pots filled with sieved sand, placed under natural conditions. Ten days after germination, the seedlings were carefully transplanted into pots containing a 2:1 mixture of soil and sand, which provided an optimal growth medium for plant growth. At 10 days’ post-germination, the rice and sesame seedlings grew 3–4 cm in height, sorghum was 4–6 cm, Egyptian pea 3–4 cm, and the Mexican hat plant 2–3 cm tall. Fungal inoculation with *F. mosseae* was performed during transplantation to evaluate its effects on plant growth and productivity. *F. mosseae* was inoculated into the growth medium twice, once with liquid culture and later with solid inoculum in growth medium (soil+ sand mixture), to test its effect on plant growth and output.

### 2.2 Inoculation with fungal strain

A 2 mL liquid culture of *F. mosseae* was prepared containing approximately 5 × 10^5^ spores/mL for fungal inoculation. Inoculum was carefully applied directly to the roots of each plant during transplantation, which was done after 10 days of germination. The inoculum of *F. mosseae* was propagated on white clover roots and consisted of spores, root fragments, mycelia, and soil (Liu et al., 2021; Szada-Borzyszkowska et al., 2024). Additionally, solid inoculum (of 50 g per pot) was incorporated into the soil-sand mixture (2:1 v/v) using the layering method (Panneerselvam et al., 2013). Briefly, the inoculum was placed 2 cm below the soil surface prior to transplanting to ensure proximity to developing roots. On the same day, inoculation was performed to allow for immediate contact between the fungus and plant roots.

This method aims to establish a beneficial symbiosis between the plant and fungus and increase root growth, nutrient uptake, and plant development. This dual inoculation strategy—root application of liquid spore suspension and soil incorporation of solid inoculum—maximized fungal colonization by combining direct root contact with sustained nutrient exchange via hyphal networks. No additional fertilizers were applied during the experimental period to ensure that any growth differences could be attributed primarily to the effect of AMF inoculation

### 2.3 Biomass yield

The biomass of the above and below-ground parts of the plants were carefully separated and measured 30 DAT (days after transplantation). We also recorded the dry weight of both root and shoot systems, as well as the number of leaves, plant height, and other growth parameters, to evaluate how *F. mosseae* inoculation affected overall plant development and productivity (Mena-Echevarría et al., 2024).

### 2.4 Nitrogen and phosphorus contents

The plant samples were dried and grounded, and the nitrogen content was determined in both shoot and root systems by analysis of the dried plant samples using a Kjeltec 8,400 Autoanalyzer (Foss Tecator AB, Höganäs, Sweden) (Li et al., 2018). Quantitative phosphorus content was determined by ashing a 0.5 g sample and following the method described in the original text. The phosphorus concentration was calorimetrically measured following Murphy and Riley (1962). This provided some insight into the effect of fungal inoculation on nitrogen and phosphorus uptake.

### 2.5 Chlorophyll fluorescence

The chlorophyll fluorescence parameters were measured at the end of the experiment using an IMAGIN PAM Chlorophyll fluorometer (Heinz Walz GmbH, Effeltrich, Germany). The parameters measured were as follows:

- *Fv/Fm*, which represents the maximum quantum yield of primary photochemistry;
$$\frac{F_{v}}{F_{m}}=\frac{F_{m}-F_{0}}{F_{m}}$$
- *F0*, the minimum fluorescence;
- *Fm*, the maximum fluorescence;
- *Fv* reflects the variable fluorescence (Dilawar et al., 2024) and is calculated as:
$$F_{m}-F_{0}=F_{v}$$

### 2.6 Photosynthetic rate and stomatal conductance measurement

The photosynthetic rate was measured using a portable photosynthesis system (LI-6400XT, LI-COR Biosciences, Lincoln, United States). The rate of photosynthesis was determined by measuring the exchange of CO_2_ within the leaf chamber and expressed as ml CO_2_ $m^{-2}s^{-1}$ . The formula used for the calculation is:

$$\mathrm{photosynthetic}\;\mathrm{rate}=\frac{\left(C_{a}-C_{i}\right)\times \mathrm{flow}\;\mathrm{rate}}{\mathrm{area}\;\mathrm{of}\;\mathrm{leaf}}$$

Where:

C_a_ = ambient CO_2_ concentration.

C_i_ = intercellular CO_2_ concentration.

Stomatal conductance ( $g_{8}$ ) was also measured using the LI-6400XT system and expressed in ${\mathrm{mmolm}}^{-2}s^{-1}$ . The measurement was based on the diffusion of water vapor through the stomata and was calculated using the following formula:

$$g_{8}=\frac{E}{\Delta W}$$

Where:

*E =* rate of transpiration $\mathrm{mmol}m^{-2}s^{-1.}$

ΔW = difference in water vapor concentration between the leaf interior and exterior ( $\mathrm{mmol}\;mol^{-1}$ )

### 2.7 Statistical analysis

The data for plant growth, biomass yield, and nutrient content were analyzed using IBM SPSS software version 25. The data were checked for normality of variances using the Kolmogorov–Smirnov test (Drezner et al., 2010). A paired *t*-test was used to test the differences between each plant’s inoculation treatments and the control groups. Means were compared using the paired t-test. Graphs were created using GraphPad Prism 10.4.1. This analysis aimed to evaluate the effect of *F. mosseae* inoculation on plant growth, biomass yield, and nutrient uptake across different plant species, considering AM fungal treatment as a common factor influencing these parameters.

### 2.8 Mycorrhizal colonization analysis

Root samples were collected 30 days after transplantation (DAT) to assess fungal colonization (Cesaro et al., 2020; Rodrigues and Rodrigues, 2015). Roots were carefully washed, cut into 1 cm segments, and cleared in 10% KOH at 90°C for 30 min. Cleaned roots were acidified with 1% HCl for 5 min, stained with 0.05% trypan blue in lacto glycerol at 60°C for 15 min (Phillips and Hayman, 1970), and de-stained in lacto glycerol. Stained roots were mounted on slides and observed under a compound microscope (Nikon Eclipse E200) at 200 × magnification.

## 3 Results

### 3.1 Plant growth

**Figure 1:**
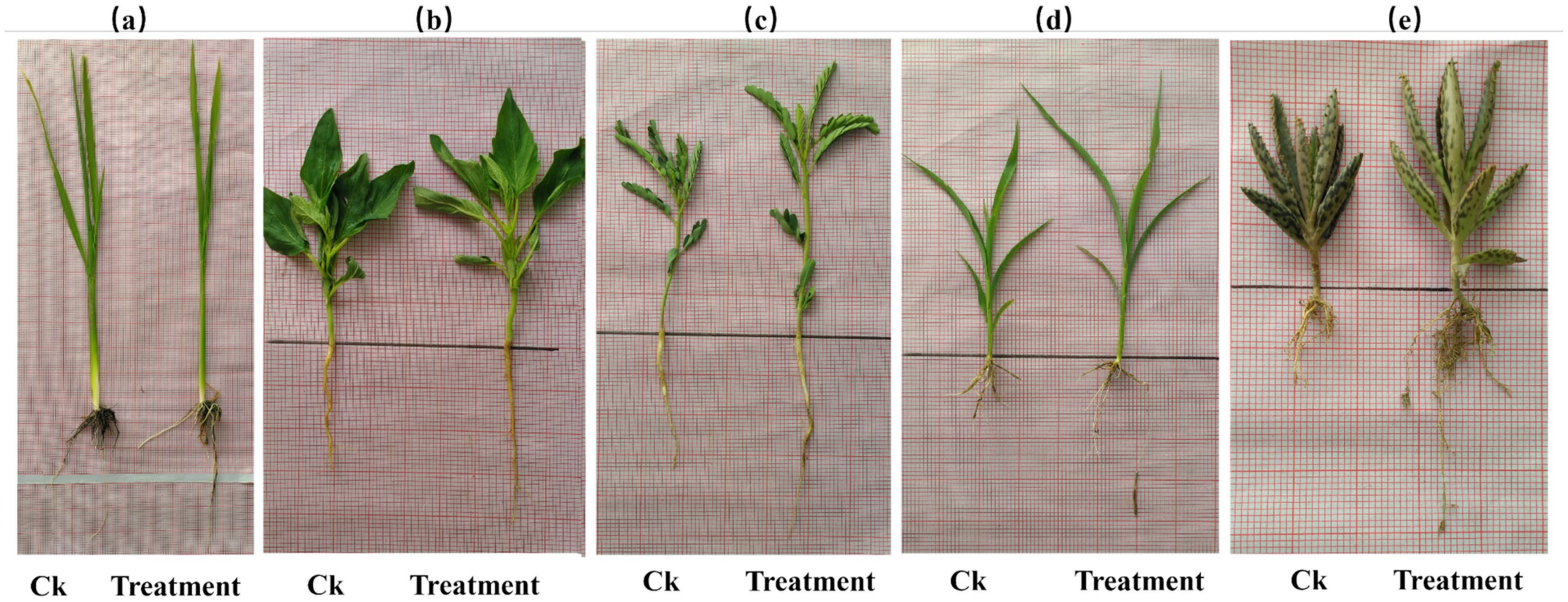
Growth comparison of root and shoot lengths between control (Ck) (left) and treatment (right) groups; **(a)** rice; **(b)** sesame; **(c)** sorghum; **(d)** Egyptian pea; and **(e)** Mexican hat. The treatment plant of every comparison group shows apparent differences in root and shoot growth.

Figure 1 shows that the treatment group significantly improved root and shoot growth in all tested plants compared to the control group. The highest root length increase was observed in sesame (66.7%), followed by sorghum (42.9%), Egyptian pea (35%), and Mexican hat plant (33.3%). The most significant shoot length increase occurred in rice (52%), followed by Sesame (50%) and Sorghum (50%).

The treatment also increased total biomass across all species (Table 2). The highest fresh biomass increase was recorded in the Egyptian pea (29%) and Mexican hat plant (31%), while the most significant dry biomass increase occurred in the Mexican hat plant (34%), followed by Egyptian pea (33%) and sesame (39%).

**Table 2:** Fresh and dry weights of different plant tissues, total biomass in control and treatment groups.

Plant	Treatment	Root fresh weight (g)	Leaf fresh weight (g)	Total fresh weight (g)	Root dry weight (g)	Leaf dry weight (g)	Total dry weight (g)	Total biomass (g)
Rice	Treatment	20.34 ± 4.56a	12.32 ± 2.34a	32.66 ± 5.98a	4.78 ± 0.92a	2.31 ± 0.46a	7.09 ± 1.35a	39.75 ± 7.33a
	Control	15.23 ± 3.12b	8.44 ± 1.14b	23.67 ± 4.12b	3.45 ± 0.85b	1.52 ± 0.32b	4.97 ± 1.12b	28.64 ± 5.24b
Sesame	Treatment	18.45 ± 3.56a	11.89 ± 2.12a	30.34 ± 5.45a	3.92 ± 0.85a	2.01 ± 0.34a	5.93 ± 1.12a	36.27 ± 6.57a
	Control	14.12 ± 2.43b	9.26 ± 1.05b	23.38 ± 3.58b	2.87 ± 0.78b	1.38 ± 0.27b	4.25 ± 1.05b	27.63 ± 4.63b
Egyptian pea	Treatment	21.72 ± 4.12a	14.53 ± 2.45a	36.25 ± 6.57a	4.29 ± 0.91a	2.12 ± 0.39a	6.41 ± 1.12a	42.66 ± 7.61a
	Control	17.25 ± 3.21b	10.89 ± 1.73b	28.14 ± 4.85b	3.12 ± 0.71b	1.69 ± 0.31b	4.81 ± 1.02b	32.95 ± 5.88b
Sorghum	Treatment	19.15 ± 3.61a	10.78 ± 2.02a	29.93 ± 5.74a	4.12 ± 0.85a	2.01 ± 0.39a	6.13 ± 1.12a	36.06 ± 6.97a
	Control	16.32 ± 2.98b	7.56 ± 1.06b	23.88 ± 4.04b	3.28 ± 0.74b	1.23 ± 0.31b	4.51 ± 1.05b	26.39 ± 5.14b
Mexican Hat Plant	Treatment	22.88 ± 4.14a	13.22 ± 2.48a	36.10 ± 6.53a	4.67 ± 0.93a	2.36 ± 0.42b	7.03 ± 1.20b	43.13 ± 7.70a
	Control	18.56 ± 3.24b	9.12 ± 1.11b	27.68 ± 4.35b	3.56 ± 0.79b	1.68 ± 0.32b	5.24 ± 1.07b	32.92 ± 5.65b

Data are means ± SD. A paired t-test was used at *p* ≤ 0.05. Statistically significant differences between the control and treatment groups are indicated by different letters in the same column (a, b) (mean of six plants).

In rice, the fresh biomass increased by 38%, from 23.67 grams to 32.66 grams (*p* < 0.05), while dry biomass increased by 43%, from 4.97 grams to 7.09 grams. Similarly, sesame showed a 30% increase in fresh biomass, from 23.38 grams to 30.34 grams (*p* < 0.05), and a 39% increase in dry biomass, from 4.25 grams to 5.93 grams. Sorghum’s biomass increased by 36.6%, while Egyptian pea and Mexican hat plants gained 29.5 and 31%, respectively.

These findings confirmed that the *F. mosseae* inoculation treatments significantly enhanced root, shoot, and biomass accumulation in all tested plant species.

**Figure 2:**
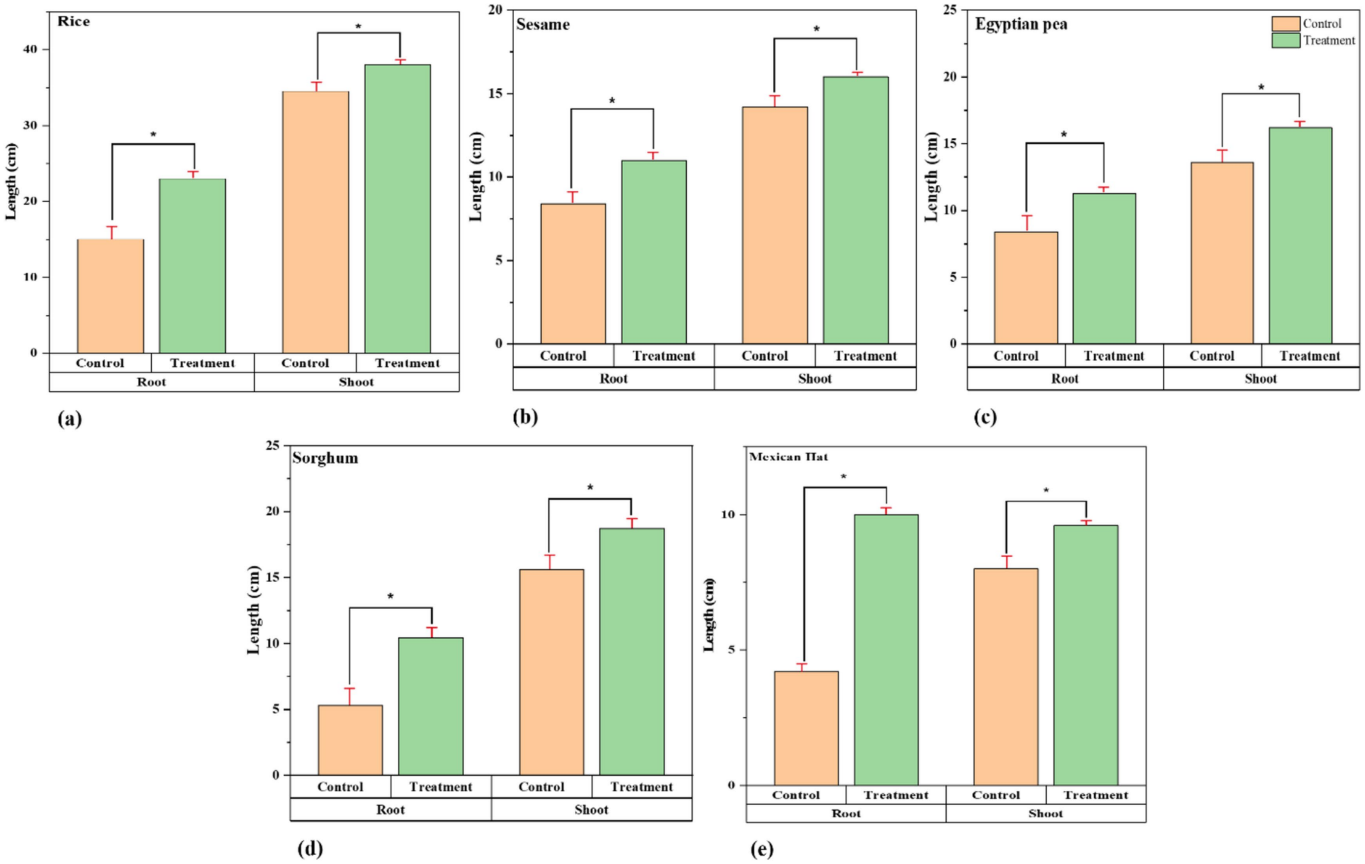
Growth comparison of root and shoot lengths between control and treatment groups. Paired t-tests revealed significant differences in root and shoot growth for all plant species (*p* < 0.05) between the control and treatment group: **(a)** Rice, **(b)** Sesame, **(c)** Sorghum, **(d)** Egyptian pea, and **(e)** Mexican hat plant. The treatment plants in every comparison group show significant differences in root and shoot growth (length). The asterisk sign represents a significant difference between the control and treatment groups.

The Figure 2 shows that root growth and treatment improvement in rice, sesame, Egyptian pea, sorghum, and Mexican Hat plants increased significantly (Supplementary Table S1). There are also exceptional results regarding the roots in the treatment group, where we see longer and thicker root systems as an indicator of better root development in the treatment group. Shoot growth also showed an increase in growth rate beyond that in the control group (see Table 3).

**Table 3:** Root nutrient concentrations (mg kg^−1^) in control and treatment groups (dry weight samples).

Plant species	Treatment	Nitrogen (N)	Phosphorus (P)	Potassium (K)	Calcium (Ca)	Magnesium (Mg)
Rice	Treatment	2.05 ± 0.13a	0.69 ± 0.13a	7.90 ± 1.44a	1.04 ± 0.12a	0.49 ± 0.06a
	Control	1.98 ± 0.15b	0.45 ± 0.14b	6.54 ± 1.53b	0.92 ± 0.09b	0.42 ± 0.05b
Sesame	Treatment	2.20 ± 0.38a	0.46 ± 0.08ab	7.12 ± 1.60a	1.19 ± 0.09a	0.51 ± 0.06a
	Control	1.87 ± 0.17b	0. 37 ± 0.12b	5.01 ± 0.84b	0.77 ± 0.08b	0.42 ± 0.07b
Egyptian pea	Treatment	2.07 ± 0.13a	0.52 ± 0.06a	6.70 ± 1.60a	0.91 ± 0.08a	0.49 ± 0.05a
	Control	1.90 ± 0.11b	0.41 ± 0.07b	5.69 ± 0.85b	0.83 ± 0.09b	0.40 ± 0.07b
Sorghum	Treatment	1.90 ± 0.10a	0.47 ± 0.09a	6.61 ± 1.50a	1.10 ± 0.09a	0.47 ± 0.06a
	Control	1.75 ± 0.08b	0.39 ± 0.08b	5.84 ± 0.76b	0.85 ± 0.06b	0.39 ± 0.05b
Mexican Hat Plant	Treatment	1.97 ± 0.08a	0.49 ± 0.07a	6.94 ± 1.60a	1.13 ± 0.34a	0.45 ± 0.06a
	Control	1.88 ± 0.10b	0.42 ± 0.08b	6.18 ± 0.73b	0.88 ± 0.11b	0.34 ± 0.03b

Data are means ± SD. A paired *t*-test was used at *p* ≤ 0.05. Statistically significant differences between the control and treatment groups are indicated by different letters in the same column (a, b) (mean of six plants).

### 3.2 Plant tissue nutrient concentrations

According to results, the plant species absorbed more root nitrogen and phosphorus through treatment with *F. mosseae* (*p* < 0.05). Sesame roots absorbed 17.6% more nitrogen after receiving the same treatment, and their nitrogen level rose from 1.87 mg/kg in controls to 2.20 mg/kg. Applying *F. mosseae* increased root nitrogen uptake by 9% in Egyptian pea seeds but only 3.5% in rice roots, from 1.98 mg/kg to 2.05 mg/kg.

The rice root phosphorus absorption reached 53% as rice plants uptake 0.45 mg/kg from the control but 0.69 mg/kg from the treated soil (*p* < 0.05). Similarly, phosphorus increased in Egyptian pea (28.6%) and Mexican hat plant (16.7%). Potassium (K) uptake was significantly higher in sesame (42%), increasing from 5.01 mg/kg to 7.12 mg/kg, and in Egyptian pea (17.8%), increased from 5.69 mg/kg to 6.70 mg/kg.

Calcium and magnesium levels increased significantly in treated plants. Treated sesame plants absorbed more calcium by 54.5%, increasing total levels from 0.77 mg/kg (control) to 1.19 mg/kg (treatment). In the Sorghum and Mexican hat plants, improvements of 29.4 and 28.4% in Ca uptake were observed, respectively. Magnesium (Mg) concentrations increased the most in the Mexican hat plant (32.4%), where Mg rose from 0.34 mg/kg to 0.45 mg/kg, followed by Egyptian pea (22.5%), increasing from 0.40 mg/kg to 0.49 mg/kg.

The leaf tissues of rice plants experienced significant increases in magnesium content in the treatment group. The rice plants showed a 24.5% magnesium concentration increase from 0.49 mg/kg control to 0.61 mg/kg treatment levels (*p* < 0.05), followed by Mexican hat plant (20.4%) and sorghum (10.6%) (Table 4).

**Table 4:** Leaves nutrient concentrations (mg kg^−1^) in control and treatment groups (dry weight samples).

Plant species	Treatment	Nitrogen (N)	Phosphorus (P)	Potassium (K)	Calcium (Ca)	Magnesium (Mg)
Rice	Treatment	4.12 ± 0.09a	0.51 ± 0.01a	8.08 ± 0.22a	1.35 ± 0.09a	0.61 ± 0.07a
	Control	3.76 ± 0.73b	0.41 ± 0.03b	7.69 ± 0.20b	1.17 ± 0.08b	0.49 ± 0.09b
Sesame	Treatment	4.12 ± 0.08a	0.51 ± 0.09a	8.14 ± 0.42a	1.26 ± 0.12a	0.52 ± 0.07a
	Control	3.98 ± 0.13b	0.42 ± 0.06b	7.77 ± 0.19b	1.08 ± 0.15b	0.47 ± 0.08b
Egyptian pea	Treatment	4.12 ± 0.09a	0.51 ± 0.03a	8.08 ± 0.23a	1.35 ± 0.09a	0.55 ± 0.03a
	Control	3.76 ± 0.73b	0.41 ± 0.03b	7.69 ± 0.48b	1.17 ± 0.08b	0.51 ± 0.05b
Sorghum	Treatment	4.05 ± 0.07a	0.48 ± 0.04a	8.22 ± 0.16a	1.31 ± 0.22a	0.52 ± 0.04a
	Control	3.85 ± 0.09b	0.41 ± 0.07b	7.72 ± 0.32b	1.09 ± 0.11b	0.47 ± 0.09b
Mexican Hat Plant	Treatment	4.12 ± 0.01a	0.52 ± 0.04a	8.10 ± 0.19a	1.28 ± 0.16a	0.53 ± 0.11a
	Control	3.92 ± 0.12b	0.47 ± 0.01b	7.94 ± 0.24b	0.92 ± 0.36b	0.44 ± 0.13b

Data are means ± SD. A paired *t-*test was used at *p* ≤ 0.05. Statistically significant differences between the control and treatment groups are indicated by different letters in the same column (a, b) (mean of six plants).

### 3.3 Physiological performance (photosynthetic rate, chlorophyll content, stomatal conductance)

**Figure 3:**
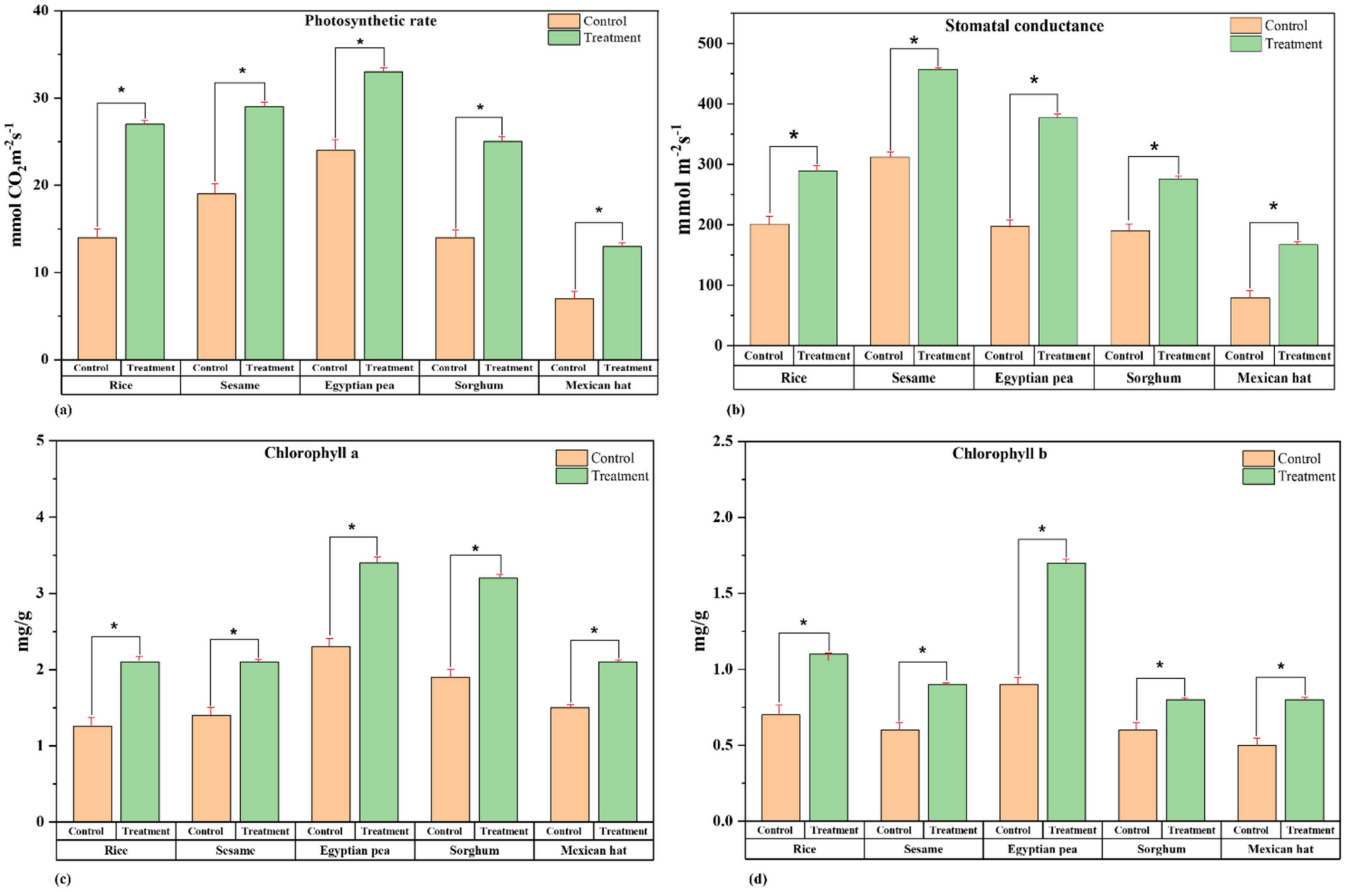
Physiochemical performance comparison between control and treatment groups. Paired t-tests revealed significant differences in all parameters between control and treatment groups: **(a)** Photosynthetic rate $\left(mmCO_{2}m^{-2}s^{-1}\right)$ in rice, sesame, Egyptian pea, sorghum, and Mexican hat plant. **(b)** Stomatal conductance $\left(\mathrm{mm}\;m^{-2}s^{-1}\right)$ in rice, sesame, Egyptian pea, sorghum, and Mexican hat plant. **(c)** *Chlorophyll is a concentration (mg/g)* in rice, sesame, Egyptian pea, sorghum, and Mexican hat plants. **(d)** *Chlorophyll b* concentration (mg/g) in rice, sesame, Egyptian pea, sorghum, and Mexican hat plant. The asterisk sign represents a significant difference between the control and treatment groups.

The photosynthetic rate, chlorophyll content, and stomatal opening were measured to evaluate the treatment’s effect on plant physiological performance. The photosynthetic rate (Figure 3a) (Supplementary Table S2) significantly increased in all treatment plants, with the highest increases recorded in Egyptian pea (45%), where the rate rose from 22 mmol CO₂ m^−2^ s^−1^ (control) to 32.1 mmol CO₂ m^−2^ s^−1^ (treatment), This was followed by sorghum (27.8%), sesame (25%), and Mexican hat plant (25%), while the lowest increase was observed in rice (21.4%) (Figure 3).

Stomatal conductance improved significantly (Figure 3b) (Supplementary Table S3), with the highest increase in sesame (71.4%), where conductance increased from 280 mmol m^−2^ s^−1^ (control) to 480 mmol m^−2^ s^−1^ (treatment). This was followed by Egyptian pea (50%), sorghum (40%), and Mexican hat plant (40%), while the lowest increase was observed in rice (23.3%). The treatment significantly increased Chlorophyll a, with the highest rise in Egyptian pea (39.1%), followed by Sesame (35.7%), Rice (33.3%), Sorghum (32.1%), and Mexican hat plant (30%). Chlorophyll b increased most in Egyptian pea (150.8%), followed by sorghum (100%), Mexican hat plant (50%), rice (50%), and sesame (44.4%) (Figures 3b,c) (Supplementary Tables S4, S5).

These findings indicate that *F. mosseae* inoculation significantly enhances plant physiological performance, with Mexican hat plant and Egyptian pea showing the strongest responses across multiple parameters.

### 3.4 Mycorrhizal colonization

**Figure 4:**
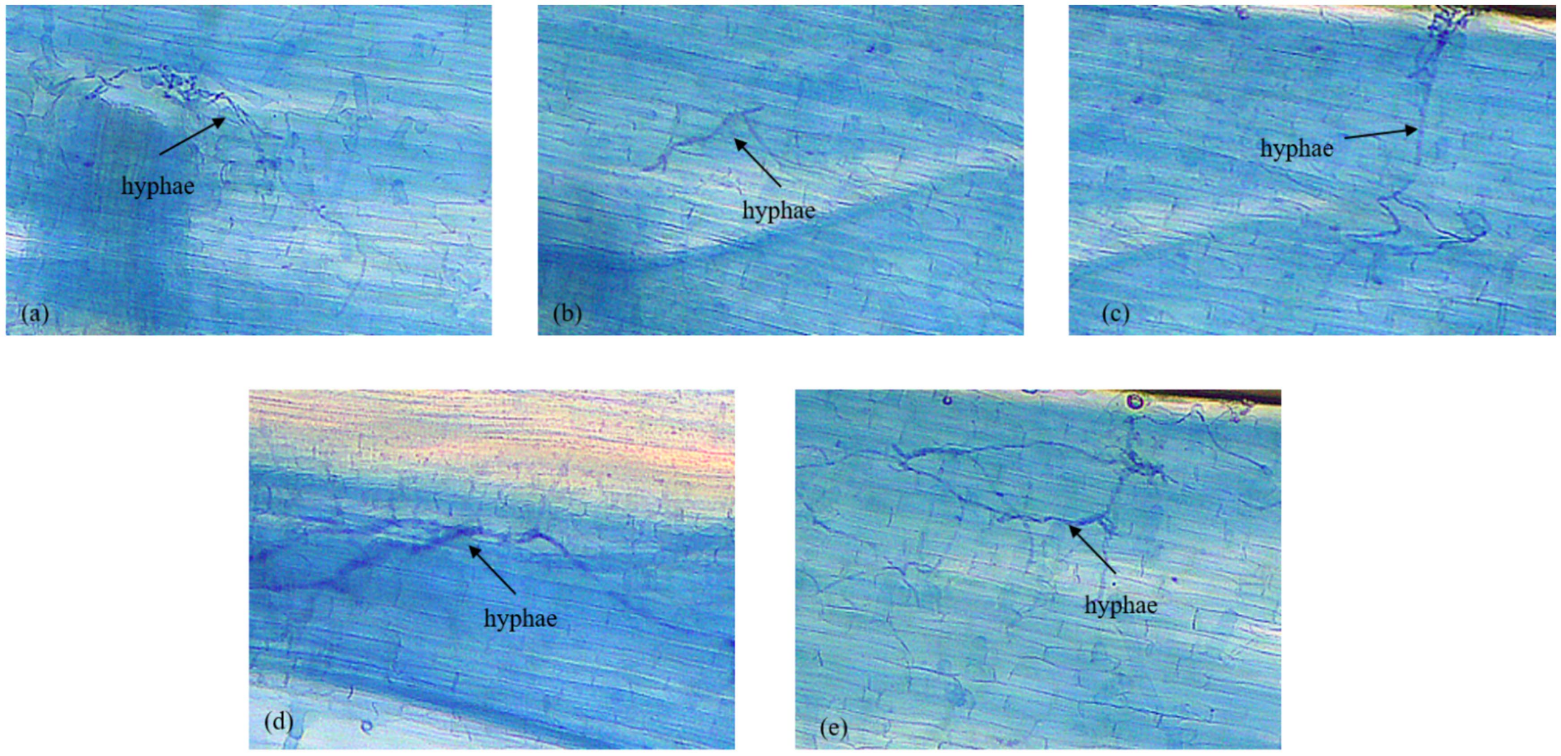
Development of mycorrhizal growth (hyphae) in plant treated with AMF *F. mosseae*. **(a)** Mexican hat plant; **(b)** rice; **(c)** sesame; **(d)** sorghum; and **(e)** Egyptian pea.

The study found that *F. mosseae* effectively colonized the root systems of all treatment plants (Figure 4). Characteristic mycorrhizal hyphae are shown in Figure 4.

## 4 Discussion

This study reveals the potential of *F. mosseae* as a plant growth-promoting fungus (PGPF) that profoundly impacts nutrient uptake and physiological performance in a range of plant species. The results indicated that fungal inoculation was incredibly beneficial for biomass accumulation, nutrient concentrations, and stress resilience, which validates the current avalanche of research on the effects of AMF.

The increase in biomass yield across all tested plant species was most notable in rice and sesame because of increased nutrient acquisition mediated by *F. mosseae*. One of the primary mechanisms by which *F. mosseae* promotes plant growth is improving nutrient availability (Ain et al., 2024). Specifically, the fungus enhances the ability of plants to access nutrients in the soil, particularly those that are less mobile, such as phosphorus and nitrogen (Sanhueza et al., 2024). This might be due to its ability to extend the root zone by establishing extensive hyphal networks that effectively increase the fungus’s access to immobile nutrients, such as phosphorus and potassium (Ain et al., 2024). The significant improvements in N and P uptake in Table 2 agree with those of previous studies (Bisht and Garg, 2022; Hussain et al., 2021; Zhu et al., 2024), demonstrating that AMF improves the uptake of soil-bound nutrients and enhances nutrient use efficiency (Table 2). Specifically, this interplay between fungal colonization and root growth promotion is significant in species with more extensive root systems, including rice and Mexican hat plants.

Interestingly, the patterns observed in the uptake of nutrients in this study also help to explain how different plant species respond to fungal inoculation. In the case of the Mexican hat plant, a species known for its tolerance to abiotic stress, Mg (0.45 mg/kg) and Ca (1.13 mg/kg) uptake enormously increased compared to the control (0.34 mg/kg, 0.88 mg/kg), respectively. Concerning the reported improvements in nitrogen, potassium, calcium, and magnesium uptake, and the assertion that *F. mosseae* typically excels in phosphorus uptake only: While it is true that AM fungi are renowned for enhancing phosphorus uptake, recent literature has also shown that *F. mosseae* can influence the uptake of other nutrients (Shi et al., 2021; Wu et al., 2024). In our experiments, the enhanced uptake of N, K, Ca, and Mg may be partly attributed to the fungus-induced improvements in root architecture and overall plant vigor, which in turn facilitate better soil exploration and nutrient acquisition. Our results implied that AMF inoculation facilitates plant growth by promoting the uptake of secondary macronutrients essential for plant physiological stability. This is consistent with studies on AMF-induced stress tolerance, in which the pivotal role of Mg in photosynthetic efficiency and chlorophyll synthesis has been demonstrated (Hamzehzadeh et al., 2024; Wahab et al., 2023; Ye et al., 2019). While this study primarily focused on the effects of *F. mosseae* on nutrient uptake and root development, it is possible that the fungus also influences plant growth through the production of growth-regulating hormones such as auxins and cytokinins (Yasmeen et al., 2024). In other studies involving mycorrhizal fungi, these hormones have been shown to enhance root development and overall plant vigor (Zhang et al., 2019). Although hormonal regulation was not directly assessed in this study, the significant improvements in biomass and nutrient concentrations observed in the treatment groups suggest that hormonal interactions may also play a role in the growth-promoting effects of *F. mosseae*.

Other measurements of chlorophyll fluorescence and photosynthetic performance also support the positive impact of *F. mosseae* on plant health. Previous research confirmed that AMF can alleviate oxidative stress and improve photosynthetic apparatus under suboptimal conditions (Chauhan et al., 2022; Mo et al., 2016). However, differences in chlorophyll content and photosynthetic rates among species observed in this study demonstrate the complexity of AMF-plant interactions and underscore the necessity of species-specific application strategies.

As shown in Figure 2, the results of root development revealed that inoculated plants developed vigorous root systems with greater length and density. This improved root system development can be attributed to mycorrhizal colonization of the roots by *F. mosseae* (Figure 4) (Huang et al., 2019). The fungus facilitates nutrient exchange by enhancing the root surface area and improving the ability of plants to absorb water and nutrients. This process is a well-documented mechanism through which AMF promotes plant growth, particularly in nutrient-poor soils (Chen et al., 2017). This is of great significance given the importance of roots for nutrient acquisition in nutrient-poor soils and for species that use extensive root networks, such as Egyptian peas, Mexican hat plants, and sorghum. *F. mosseae* has excellent potential as a biofertilizer in regions with degraded soils or low access to chemical fertilizers because the synergy of *F. mosseae* on root architecture and nutrient uptake efficiency has been reaffirmed.

One of these promising findings also raises intriguing patterns that warrant further investigation. For example, although *F. mosseae* significantly increased most species’ growth and nutrient uptake, the magnitude of the increase differed. The source of this variability may be differences in the morphology of the root, compatibility with fungal symbionts, or inherent nutrient requirements of a particular organism (Bever, 2015). Furthermore, slight differences in Mg uptake between species indicate that while fungal inoculation may differentially partition nutrients, the existing literature needs to explore this topic.

The present study presents the ecological potential and potential for agronomic use of *F. mosseae*; further work is needed to develop an appropriate application. Co-inoculation of AMF with other plant growth-promoting microorganisms (PGPMs) may synergistically affect soil respiration, nutrient cycling, and plant resistance. In addition, long-term field trials are necessary to evaluate the sustainability and scalability of integrating AMF in various cropping systems.

Finally, the results of the investigations showed that *F. mosseae* has significant potential for stimulating the growth, nutrient uptake, and physiological performance of many plant species. Such findings indicate that it can be integrated into agricultural practices as an eco-friendly farming mode instead of chemical use. *F. mosseae* provides a pathway towards sustainable agricultural systems through improvements in crop productivity without loss of soil health. Nevertheless, it will not reveal the full potential of its interactions with plants, soils, and other microbial communities under changing environments.

## 5 Conclusion and future perspective

Our study provides clear evidence that *Funneliformis mosseae* inoculation significantly improves plant growth, nutrient uptake, and physiological performance across all tested species. The most notable improvements were seen in rice, which showed a 43% increase in dry biomass, and sesame, which demonstrated a 71.4% improvement in stomatal conductance, might be due to better water uptake and hormonal improvement. We observed particularly strong enhancements in phosphorus uptake in rice (53% increase) and magnesium accumulation in the Mexican hat plant (32.4% increase). Microscopic examination confirmed successful fungal colonization in all treated plants directly linking these benefits to the AMF symbiosis. These results indicate that *F. mosseae* has the potential to significantly enhance plant growth, nutrient uptake, and physiological performance in a variety of economically and ecologically essential crops. This symbiotic fungus improves biomass yield, nutrient acquisition, and stress resilience and provides a sustainable alternative to chemical fertilizers to remedy problems of soil degradation, environmental pollution, and food security. Future research studies are needed on how plant growth responds when recommended dose fertilizer (RDF) applications are supplemented with Arbuscular Mycorrhizal Fungi inoculation. Research with different fertilizer recommendations will show how well AMF works alongside regular fertilization methods for plant development. Prospective research should be focused on learning how AMF works with fertilizers to help plants grow better without depending more on chemical fertilizers. Future work should focus on long-term field studies to validate its efficacy under diverse agroecological conditions, investigate its interaction with other microbial inoculants, and optimize species-specific applications. Improved integration of *F. mosseae* into modern agricultural practices is an essential first step towards developing sustainable farming systems that promote healthier soils, minimize environmental footprints, and build resilience to the impact of climate change to guarantee food security and sustainability for the world.

## Data Availability

The original contributions presented in the study are included in the article/Supplementary material, further inquiries can be directed to the corresponding author.

## References

[ref1] Abrar M. Zhu Y. Rehman M. M. U. Batool A. Duan H.-X. Ashraf U.. (2024). Functionality of arbuscular mycorrhizal fungi varies across different growth stages of maize under drought conditions. Plant Physiol. Biochem. 213:108839. doi: 10.1016/j.plaphy.2024.108839, PMID: 38879986

[ref9001] Agnihotri R. Bharti A. Ramesh A. Prakash A. Sharma M. P. (2021). Glomalin related protein and C16: 1ω5 PLFA associated with AM fungi as potential signatures for assessing the soil C sequestration under contrasting soil management practices. Eur. J. Soil Biol. 103:103286. doi: 10.1016/j.ejsobi.2021.103286

[ref2] Ain Q. U. Hussain H. A. Zhang Q. Maqbool F. Ahmad M. Mateen A.. (2024). Coordinated influence of Funneliformis mosseae and different plant growth-promoting bacteria on growth, root functional traits, and nutrient acquisition by maize. Mycorrhiza 34, 477–488. doi: 10.1007/s00572-024-01165-5, PMID: 39115556

[ref3] Baweja P. Kumar S. Kumar G. (2020). Fertilizers and pesticides: their impact on soil health and environment. Soil Health. 59, 265–285. doi: 10.1007/978-3-030-44364-1_15

[ref4] Bennett A. E. Groten K. (2022). The costs and benefits of plant–arbuscular mycorrhizal fungal interactions. Annu. Rev. Plant Biol. 73, 649–672. doi: 10.1146/annurev-arplant-102820-124504, PMID: 35216519

[ref5] Bever J. D. (2015). Preferential allocation, physio-evolutionary feedbacks, and the stability and environmental patterns of mutualism between plants and their root symbionts. New Phytol. 205, 1503–1514. doi: 10.1111/nph.13239, PMID: 25561086

[ref6] Bisht A. Garg N. (2022). AMF species improve yielding potential of cd stressed pigeonpea plants by modulating sucrose-starch metabolism, nutrients acquisition and soil microbial enzymatic activities. Plant Growth Regul. 96, 409–430. doi: 10.1007/s10725-021-00791-9

[ref7] Cesaro P. Massa N. Cantamessa S. Todeschini V. Bona E. Berta G.. (2020). Tomato responses to Funneliformis mosseae during the early stages of arbuscular mycorrhizal symbiosis. Mycorrhiza 30, 601–610. doi: 10.1007/s00572-020-00973-9, PMID: 32621137

[ref9002] Chandwani S. Maiti S. Amaresan N. (2023). Fungal mycorrhizae from plants roots: Functions and molecular interactions. In Microbial Symbionts (pp. 133–160). Academic Press.

[ref8] Chauhan S. Mahawar S. Jain D. Udpadhay S. K. Mohanty S. R. Singh A.. (2022). Boosting sustainable agriculture by arbuscular mycorrhiza under stress condition: mechanism and future prospective. Biomed. Res. Int. 2022:5275449. doi: 10.1155/2022/5275449, PMID: 36619307 PMC9815931

[ref9] Chen M. Yang G. Sheng Y. Li P. Qiu H. Zhou X.. (2017). Glomus mosseae inoculation improves the root system architecture, photosynthetic efficiency and flavonoids accumulation of liquorice under nutrient stress. Front. Plant Sci. 8:931. doi: 10.3389/fpls.2017.00931, PMID: 28638391 PMC5461296

[ref10] Davoudpour Y. Schmidt M. Calabrese F. Richnow H. H. Musat N. (2020). High resolution microscopy to evaluate the efficiency of surface sterilization of *Zea Mays* seeds. PLoS One 15:e0242247. doi: 10.1371/journal.pone.0242247, PMID: 33253171 PMC7703986

[ref9003] Dahiya G. Bhardwaj K. K. Ahlawat I. Singh C. Devi S. Kumar S.. (2022). Glomalin: a miracle protein for carbon sequestration. Int. J. Plant Soil Sci., 2022, 80–86.

[ref11] Dilawar N. Hamayun M. Iqbal A. Lee B. Ali S. Ahmad A.. (2024). Rhizofungus aspergillus terreus mitigates heavy metal stress-associated damage in *Triticum aestivum* L. Plan. Theory 13:2643. doi: 10.3390/plants13182643, PMID: 39339618 PMC11435276

[ref12] Drezner Z. Turel O. Zerom D. (2010). A modified Kolmogorov–Smirnov test for normality. Commun. Stat. Simul. Comput. 39, 693–704. doi: 10.1080/03610911003615816

[ref13] Gamage A. Gangahagedara R. Gamage J. Jayasinghe N. Kodikara N. Suraweera P.. (2023). Role of organic farming for achieving sustainability in agriculture. Farm. Syst. 1:100005. doi: 10.1016/j.farsys.2023.100005

[ref14] Hamzehzadeh H. Abbaspour H. Safipour Afshar A. Hamdi S. M. M. (2024). AMF-mediated salinity adaptation in pistachio plants: photosynthetic efficiency and ionic balance. Biologia 80, 1–17. doi: 10.1007/s11756-024-01810-6

[ref15] Huang L. Chen D. Zhang H. Song Y. Chen H. Tang M. (2019). Funneliformis mosseae enhances root development and Pb phytostabilization in *Robinia pseudoacacia* in Pb-contaminated soil. Front. Microbiol. 10:2591. doi: 10.3389/fmicb.2019.02591, PMID: 31781076 PMC6861453

[ref16] Hussain S. Sharif M. Ahmad W. (2021). Selection of efficient phosphorus solubilizing bacteria strains and mycorrhizea for enhanced cereal growth, root microbe status and N and P uptake in alkaline calcareous soil. Soil Sci. Plant Nutr. 67, 259–268. doi: 10.1080/00380768.2021.1904793

[ref17] John D. A. Babu G. R. (2021). Lessons from the aftermaths of green revolution on food system and health. Front. Sustain. Food Syst. 5:644559. doi: 10.3389/fsufs.2021.644559, PMID: 34212131 PMC7611098

[ref18] Khan W. Zhu Y. Khan A. Zhao L. Yang Y.-M. Wang N.. (2024). Above-and below-ground feedback loop of maize is jointly enhanced by plant growth-promoting rhizobacteria and arbuscular mycorrhizal fungi in drier soil. Sci. Total Environ. 917:170417. doi: 10.1016/j.scitotenv.2024.170417, PMID: 38280611

[ref19] Li J. Shi Y. Veeranampalayam-Sivakumar A.-N. Schachtman D. P. (2018). Elucidating sorghum biomass, nitrogen and chlorophyll contents with spectral and morphological traits derived from unmanned aircraft system. Front. Plant Sci. 9:1406. doi: 10.3389/fpls.2018.01406, PMID: 30333843 PMC6176777

[ref20] Liu J. Ge X. Fan X. Liu H. Gao Y. Ren A. (2021). The inhibitory effect of endophyte-infected tall fescue on white clover can be alleviated by Glomus mosseae instead of rhizobia. Microorganisms 9:109. doi: 10.3390/microorganisms9010109, PMID: 33466333 PMC7824791

[ref21] Malgioglio G. Rizzo G. F. Nigro S. Lefebvre du Prey V. Herforth-Rahmé J. Catara V.. (2022). Plant-microbe interaction in sustainable agriculture: the factors that may influence the efficacy of PGPM application. Sustain. For. 14:2253. doi: 10.3390/su14042253

[ref22] Mena-Echevarría A. Ramírez-Tobias H. M. Méndez-Cortés H. Rojas-Velázquez Á. N. López-Palacios C. Hipólito-Piedras R. P. (2024). The origin and type of inoculum determine the effect of Arbuscular Mycorrhizal Fungi on tomato under different irrigation regimes. Agronomy 14:1687. doi: 10.3390/agronomy14081687

[ref23] Mo Y. Wang Y. Yang R. Zheng J. Liu C. Li H.. (2016). Regulation of plant growth, photosynthesis, antioxidation and osmosis by an arbuscular mycorrhizal fungus in watermelon seedlings under well-watered and drought conditions. Front. Plant Sci. 7:644. doi: 10.3389/fpls.2016.00644, PMID: 27242845 PMC4862978

[ref24] Murphy J. Riley J. P. (1962). A modified single solution method for the determination of phosphate in natural waters. Anal. Chim. Acta 27, 31–36. doi: 10.1016/S0003-2670(00)88444-5

[ref25] Panneerselvam P. Saritha B. Mohandas S. Upreti K. K. Poovarasan S. Sulladmath V. V.. (2013). Effect of mycorrhiza-associated bacteria on enhancing colonization and sporulation of Glomus mosseae and growth promotion in sapota (*Manilkara achras* (mill) Forsberg) seedlings. Biol. Agric. Hortic. 29, 118–131. doi: 10.1080/01448765.2013.779076

[ref9004] Phillips J. M. Hayman D. S. (1970). Improved procedures for clearing roots and staining parasitic and vesicular-arbuscular mycorrhizal fungi for rapid assessment of infection. Transactions of the British mycological Society, 55:158-IN18.

[ref26] Rehman M. M. U. Zhu Y. Abrar M. Khan W. Wang W. Iqbal A.. (2024). Moisture-and period-dependent interactive effects of plant growth-promoting rhizobacteria and AM fungus on water use and yield formation in dryland wheat. Plant Soil 502, 149–165. doi: 10.1007/s11104-022-05641-9

[ref27] Rodrigues K. M. Rodrigues B. F. (2015). Endomycorrhizal association of Funneliformis mosseae with transformed roots of *Linum usitatissimum*: germination, colonization, and sporulation studies. Mycology 6, 42–49. doi: 10.1080/21501203.2015.1024777, PMID: 26000198 PMC4409042

[ref28] Samuel S. S. Veeramani A. (2021). “Advantages of arbuscular mycorrhizal fungi (AMF) production for the profitability of agriculture and biofertilizer industry” in Mycorrhizal Fungi-utilization in agriculture and industry. ed. Radhakrishnan R. (UK: IntechOpen), 31–46.

[ref29] Sanhueza T. Hernández I. Sagredo-Sáez C. Villanueva-Guerrero A. Alvarado R. Mujica M. I.. (2024). Juvenile plant–microbe interactions modulate the adaptation and response of forest seedlings to rapid climate change. Plan. Theory 13:175. doi: 10.3390/plants13020175, PMID: 38256729 PMC10819047

[ref30] Sharma A. Kumar V. Shahzad B. Tanveer M. Sidhu G. P. S. Handa N.. (2019). Worldwide pesticide usage and its impacts on ecosystem. SN Appl. Sci. 1, 1–16. doi: 10.1007/s42452-019-1485-1, PMID: 40201927

[ref31] Shattuck A. Werner M. Mempel F. Dunivin Z. Galt R. (2023). Global pesticide use and trade database (GloPUT): new estimates show pesticide use trends in low-income countries substantially underestimated. Glob. Environ. Chang. 81:102693. doi: 10.1016/j.gloenvcha.2023.102693

[ref32] Shi S. Luo X. Wen M. Dong X. Sharifi S. Xie D.. (2021). Funneliformis mosseae improves growth and nutrient accumulation in wheat by facilitating soil nutrient uptake under elevated CO2 at daytime, not nighttime. J. Fungi 7:458. doi: 10.3390/jof7060458, PMID: 34200509 PMC8229587

[ref33] Si Y. Haxim Y. Wang L. (2022). Optimum sterilization method for *in vitro* cultivation of dimorphic seeds of the succulent halophyte Suaeda aralocaspica. Horticulturae 8:289. doi: 10.3390/horticulturae8040289

[ref34] Szada-Borzyszkowska A. Krzyżak J. Rusinowski S. Magurno F. Pogrzeba M. (2024). Inoculation with Arbuscular Mycorrhizal Fungi supports the uptake of macronutrients and promotes the growth of *Festuca ovina* L. and *Trifolium medium* L., a candidate species for green urban infrastructure. Plan. Theory 13:2620. doi: 10.3390/plants13182620, PMID: 39339595 PMC11434852

[ref35] Tang B. Man J. Lehmann A. Rillig M. C. (2023). Arbuscular mycorrhizal fungi benefit plants in response to major global change factors. Ecol. Lett. 26, 2087–2097. doi: 10.1111/ele.14320, PMID: 37794719

[ref36] Wahab A. Muhammad M. Munir A. Abdi G. Zaman W. Ayaz A.. (2023). Role of arbuscular mycorrhizal fungi in regulating growth, enhancing productivity, and potentially influencing ecosystems under abiotic and biotic stresses. Plan. Theory 12:3102. doi: 10.3390/plants12173102, PMID: 37687353 PMC10489935

[ref37] Wang F. Adams C. A. Yang W. Sun Y. Shi Z. (2020). Benefits of arbuscular mycorrhizal fungi in reducing organic contaminant residues in crops: implications for cleaner agricultural production. Crit. Rev. Environ. Sci. Technol. 50, 1580–1612. doi: 10.1080/10643389.2019.1665945

[ref38] Wu Y. Sun Z. Liu R. Cai B. (2024). Funneliformis mosseae enhances the function of C, N and P cycling Bacteria in continuous soybean rhizosphere soil. J. Soil Sci. Plant Nutr. 24, 8263–8279. doi: 10.1007/s42729-024-02112-1, PMID: 40201927

[ref39] Yasmeen T. Haidar W. Syrish A. Rizwan M. Arif M. S. Shahzad S. M.. (2024). “Endophytic fungi: plant growth-promoting phytohormones and their potential application” in Fungal secondary metabolites. ed. Abd-Elsalam K. A. Mohame H. I. (Amsterdam, Netherlands: Elsevier), 57–72.

[ref40] Ye L. Zhao X. Bao E. Cao K. Zou Z. (2019). Effects of arbuscular mycorrhizal fungi on watermelon growth, elemental uptake, antioxidant, and photosystem II activities and stress-response gene expressions under salinity-alkalinity stresses. Front. Plant Sci. 10:863. doi: 10.3389/fpls.2019.00863, PMID: 31333702 PMC6616249

[ref41] Zhang F. Wang P. Zou Y.-N. Wu Q.-S. Kuča K. (2019). Effects of mycorrhizal fungi on root-hair growth and hormone levels of taproot and lateral roots in trifoliate orange under drought stress. Arch. Agron. Soil Sci. 65, 1316–1330. doi: 10.1080/03650340.2018.1563780

[ref42] Zhu Z. Ju S. Li P. Li L. Yang Z. (2024). Study on the synergistic effects of siderophore bacteria and arbuscular mycorrhizal fungi on the improvement of iron nutrition in Cinnamomum camphor. J. Plant Nutr. 5, 1–13. doi: 10.1080/01904167.2024.2414752

