# Supplementary material for: Growth-promoting effects of arbuscular mycorrhizal fungus Funneliformis mosseae in rice, sesame, sorghum, Egyptian pea and Mexican hat plant
Source: Front Microbiol. 2025 Apr 28;16:1549006. doi: 10.3389/fmicb.2025.1549006 (PMC12066788; doi:10.3389/fmicb.2025.1549006)
Supplement: Supplementary file 1 [file Data_Sheet_1.zip › Supplmentary Tables/Table S1.docx]

**Table S1**

**Growth Parameters Length (Root & Shoot)**

**A-Rice- root**

| Paired t test |  |
| --- | --- |
| P value | <0.0001 |
| P value summary | **** |
| Significantly different (P < 0.05)? | Yes |
| One- or two-tailed P value? | Two-tailed |
| t, df | t=47.12, df=5 |
| Number of pairs | 6 |

**B-Rice-shoot**

| Paired t test |  |
| --- | --- |
| P value | <0.0001 |
| P value summary | **** |
| Significantly different (P < 0.05)? | Yes |
| One- or two-tailed P value? | Two-tailed |
| t, df | t=32.56, df=5 |
| Number of pairs | 6 |

**C-Sesame-root**

| Paired t test |  |
| --- | --- |
| P value | <0.0001 |
| P value summary | **** |
| Significantly different (P < 0.05)? | Yes |
| One- or two-tailed P value? | Two-tailed |
| t, df | t=31.97, df=5 |
| Number of pairs | 6 |

**D-Sesame-shoot**

| Paired t test |  |
| --- | --- |
| P value | <0.0001 |
| P value summary | **** |
| Significantly different (P < 0.05)? | Yes |
| One- or two-tailed P value? | Two-tailed |
| t, df | t=11.82, df=5 |
| Number of pairs | 6 |

**E-Egyptian Pea-Root**

| P value | <0.0001 |
| --- | --- |
| P value summary | **** |
| Significantly different (P < 0.05)? | Yes |
| One- or two-tailed P value? | Two-tailed |
| t, df | t=32.42, df=5 |
| Number of pairs | 6 |

**F-Egyptian Pea-shoot**

| Paired t test |  |
| --- | --- |
| P value | <0.0001 |
| P value summary | **** |
| Significantly different (P < 0.05)? | Yes |
| One- or two-tailed P value? | Two-tailed |
| t, df | t=35.00, df=5 |
| Number of pairs | 6 |

**G-Sorghum-Root**

| Paired t test |  |
| --- | --- |
| P value | <0.0001 |
| P value summary | **** |
| Significantly different (P < 0.05)? | Yes |
| One- or two-tailed P value? | Two-tailed |
| t, df | t=83.52, df=5 |
| Number of pairs | 6 |

**H-Sorghum –Shoot**

| Paired t test |  |
| --- | --- |
| P value | <0.0001 |
| P value summary | **** |
| Significantly different (P < 0.05)? | Yes |
| One- or two-tailed P value? | Two-tailed |
| t, df | t=29.12, df=5 |
| Number of pairs | 6 |

**I-Mexican Hat Plant-Root**

| Paired t test |  |
| --- | --- |
| P value | <0.0001 |
| P value summary | **** |
| Significantly different (P < 0.05)? | Yes |
| One- or two-tailed P value? | Two-tailed |
| t, df | t=52.59, df=5 |
| Number of pairs | 6 |

**J-Mexican Hat Plant-Shoot**

| Paired t test |  |
| --- | --- |
| P value | <0.0001 |
| P value summary | **** |
| Significantly different (P < 0.05)? | Yes |
| One- or two-tailed P value? | Two-tailed |
| t, df | t=47.80, df=5 |
| Number of pairs | 6 |
