# Supplementary material for: Growth-promoting effects of arbuscular mycorrhizal fungus Funneliformis mosseae in rice, sesame, sorghum, Egyptian pea and Mexican hat plant
Source: Front Microbiol. 2025 Apr 28;16:1549006. doi: 10.3389/fmicb.2025.1549006 (PMC12066788; doi:10.3389/fmicb.2025.1549006)
Supplement: Supplementary file 1 [file Data_Sheet_1.zip › Supplmentary Tables/Table S2.docx]

**Table S2: Photosynthesis rate**

**A. Rice**

| Paired t test |  |
| --- | --- |
| P value | <0.0001 |
| P value summary | **** |
| Significantly different (P < 0.05)? | Yes |
| One- or two-tailed P value? | Two-tailed |
| t, df | t=15.92, df=5 |
| Number of pairs | 6 |

**B. Sesame**

| Paired t test |  |
| --- | --- |
| P value | 0.0002 |
| P value summary | *** |
| Significantly different (P < 0.05)? | Yes |
| One- or two-tailed P value? | Two-tailed |
| t, df | t=10.00, df=5 |
| Number of pairs | 6 |

**C. Egyptian Pea**

| Paired t test |  |
| --- | --- |
| P value | 0.0004 |
| P value summary | *** |
| Significantly different (P < 0.05)? | Yes |
| One- or two-tailed P value? | Two-tailed |
| t, df | t=8.216, df=5 |
| Number of pairs | 6 |
|  |  |

**D. Mexican hat Plant**

| Paired t test |  |
| --- | --- |
| P value | <0.0001 |
| P value summary | **** |
| Significantly different (P < 0.05)? | Yes |
| One- or two-tailed P value? | Two-tailed |
| t, df | t=18.98, df=5 |
| Number of pairs | 6 |

**E. Sorghum**

| Paired t test |  |
| --- | --- |
| P value | <0.0001 |
| P value summary | **** |
| Significantly different (P < 0.05)? | Yes |
| One- or two-tailed P value? | Two-tailed |
| t, df | t=24.60, df=5 |
| Number of pairs | 6 |
