# Supplementary material for: Growth-promoting effects of arbuscular mycorrhizal fungus Funneliformis mosseae in rice, sesame, sorghum, Egyptian pea and Mexican hat plant
Source: Front Microbiol. 2025 Apr 28;16:1549006. doi: 10.3389/fmicb.2025.1549006 (PMC12066788; doi:10.3389/fmicb.2025.1549006)
Supplement: Supplementary file 1 [file Data_Sheet_1.zip › Supplmentary Tables/Table S3.docx]

**Table S3: Stomatal Conductance**

**A. Rice**

| Paired t test |  |
| --- | --- |
| P value | <0.0001 |
| P value summary | **** |
| Significantly different (P < 0.05)? | Yes |
| One- or two-tailed P value? | Two-tailed |
| t, df | t=55.29, df=5 |
| Number of pairs | 6 |

**B. Sesame**

| Paired t test |  |
| --- | --- |
| P value | <0.0001 |
| P value summary | **** |
| Significantly different (P < 0.05)? | Yes |
| One- or two-tailed P value? | Two-tailed |
| t, df | t=27.88, df=5 |
| Number of pairs | 6 |

**C. Egyptian pea**

| Paired t test |  |
| --- | --- |
| P value | <0.0001 |
| P value summary | **** |
| Significantly different (P < 0.05)? | Yes |
| One- or two-tailed P value? | Two-tailed |
| t, df | t=34.69, df=5 |
| Number of pairs | 6 |

**D. Sorghum**

| Paired t test |  |
| --- | --- |
| P value | <0.0001 |
| P value summary | **** |
| Significantly different (P < 0.05)? | Yes |
| One- or two-tailed P value? | Two-tailed |
| t, df | t=38.04, df=5 |
| Number of pairs | 6 |

**E. Mexican Hat Plant**

| Paired t test |  |
| --- | --- |
| P value | <0.0001 |
| P value summary | **** |
| Significantly different (P < 0.05)? | Yes |
| One- or two-tailed P value? | Two-tailed |
| t, df | t=41.90, df=5 |
| Number of pairs | 6 |
